# Impact of Hyaluronic Acid on the Cutaneous T-Cell Lymphoma Microenvironment: A Novel Anti-Tumor Mechanism of Bexarotene

**DOI:** 10.3390/cancers17020324

**Published:** 2025-01-20

**Authors:** Tetsuya Ikawa, Emi Yamazaki, Ryo Amagai, Yumi Kambayashi, Mana Sekine, Takuya Takahashi, Yoshihide Asano, Taku Fujimura

**Affiliations:** Department of Dermatology, Tohoku University Graduate School of Medicine, Sendai 980-8575, Japan; tetsuya.ikawa@derma.med.tohoku.ac.jp (T.I.); hemiura0804@derma.med.tohoku.ac.jp (E.Y.); amagai@derma.med.tohoku.ac.jp (R.A.); kambayashi@derma.med.tohoku.ac.jp (Y.K.); ma.sekine@derma.med.tohoku.ac.jp (M.S.); takuya.takahashi@derma.med.tohoku.ac.jp (T.T.); yasano@derma.med.tohoku.ac.jp (Y.A.)

**Keywords:** CTCL, hyaluronic acid (HA), bexarotene, low-molecular-weight HA, high-molecular-weight HA, HAS

## Abstract

Background: Cutaneous T-cell lymphoma (CTCL) is a type of non-Hodgkin’s lymphoma that primarily affects the skin, rich in hyaluronic acid (HA). HA is a component of the extracellular matrix in the dermis and likely affects the development of CTCL, but the mechanism is poorly understood. Here we show that low-molecular-weight HA (LMWHA) possibly exacerbates CTCL, and bexarotene, already used in CTCL treatment, decreases HA production. Methods: We conducted immunohistochemistry, qRT-PCR, immunoblotting, and HA quantification using both mouse and human specimens to evaluate the impact of HA on CTCL. Additionally, we assessed the effect of bexarotene, which is already used for CTCL treatment, on HA metabolism. Results: HA expression was higher in patients’ serum and skin sections than in healthy controls. HA extracted from the skin of mice inoculated with tumors showed an increase in LMWHA. LMWHA increased lymphoma cell proliferation in vitro and accelerated tumor formation in mice in vivo. LMWHA also created a favorable environment for tumor cells by affecting fibroblasts, vascular endothelial cells, and tumor-associated macrophages. Thus, increased levels of HA, mainly LMWHA, exacerbate CTCL progression by affecting tumor cells and their microenvironment. Bexarotene treatment reduced the amount of total HA in murine tumor-inoculated skin, as well as the supernatant of cultured normal human dermal fibroblasts (NHDFs) and HuT78 cells. Detailed in vitro analyses showed that bexarotene treatment decreased HA synthase (HAS)1 and HAS2 expression in NHDFs and HAS1 and HAS3, and CEMIP expression in HuT78 cells. Chromatin immunoprecipitation assays revealed that bexarotene reduced retinoid X receptor-α binding to the *HAS1* and *HAS2* promoters in NHDFs. Conclusions: Bexarotene potentially exerts its anti-tumor effect by reducing HA levels through decreased expression of HAS. These findings provide new insights into the process of CTCL development and additional insights regarding bexarotene treatment.

## Simple Summary

The disease course of cutaneous T cell lymphoma (CTCL) is indolent, but progression increases rapidly after tumor formation. Therefore, identifying the factors that trigger tumor formation is important. CTCL primarily affects the skin, rich in hyaluronic acid (HA). HA is a component of the extracellular matrix in the dermis and likely affects the development of CTCL, but the mechanism is poorly understood. We show that low-molecular-weight HA (LMWHA) increased lymphoma cell proliferation in vitro and accelerated tumor formation in vivo by affecting cancer fibroblasts, vascular endothelial cells, and tumor-associated macrophages. Moreover, we demonstrate that bexarotene reduces LMWHA via binding of the retinoid X receptor to the HA synthase gene, providing an additional anti-tumor effect. The results of our study demonstrate that HA plays a role in the development of CTCL by affecting the tumor and its microenvironment. These findings suggest a novel therapeutic approach for cancer treatment that focuses on targeting HA.

## 1. Introduction

Cutaneous T-cell lymphoma (CTCL) is a type of T-cell lymphoma derived from the skin. Mycosis fungoides (MF) is the most common type of CTCL [1]. The disease course of MF is indolent in the early stage, but progression increases rapidly after tumor formation. Therefore, identifying the factors that trigger tumor formation is important [2].

Hyaluronic acid (HA) is a fundamental component of the extracellular matrix, which gives skin moisture, elasticity, and stability. HA is produced by membrane-bound enzymes known as HA synthase (HAS) 1-3, and in vitro studies revealed that HAS1 and HAS2 can synthesize larger polymers (greater than 2 × 10^6^ Da) than HAS3 (1 × 10^5^ to 1 × 10^6^ Da) [3]. The primary source of HA in the skin is fibroblasts, which express all of the HASs [4]. HA that is >1000 kDa is generally classified as high-molecular-weight HA (HMWHA), which exhibits immunosuppressive, anti-tumor, and antiangiogenic properties [5]. HMWHA is degraded into a smaller molecule known as low-molecular-weight HA (LMWHA) during catabolism or under conditions of inflammation, tissue damage, and tumorigenesis [5]. The classification of HA based on molecular size varies from study to study, but in this study, HA > 500 kDa was defined as HMWHA, and HA < 500 kDa was defined as LMWHA.

In the present study, we focused on catabolism of HA. The well-characterized HA-depolymerizing enzymes in the skin are hyaluronidase (HYAL) 1 and 2. HYAL2 cleaves HMWHA into fragments of approximately 20 kDa at the surface of the cell membrane, and these fragments are internalized by the lysosomal system and further degraded by HYAL1 [3]. CEMIP and CEMIP2, recently identified enzymes, are also involved in HA degradation [6]. The activity of the latter CEMIP/CEMIP2 system leads to an accumulation of LMWHA in the extracellular space.

HA is reportedly involved in tumor progression [7]. In addition to being a component of the extracellular matrix, HA functions as a ligand that mediates signal transduction via CD44, toll-like receptor 4, and hyaluronan-mediated motility receptor (HMMR) [7]. Moreover, HA is reportedly involved in multiple types of cancer, including lymphoma. Serum HA levels are higher in patients with Hodgkin’s lymphoma, non-Hodgkin’s lymphoma, and acute leukemia, and might serve as a marker of prognosis or relapse [8,9]. Although considerable evidence suggests that HA plays a role in cancer progression, the contribution of HA in the development of CTCL has not been thoroughly investigated. Since CTCL cells developed in the skin, which is an HA-rich environment, in this study, we examined the role of HA in the CTCL tumor microenvironment.

## 2. Materials and Methods

### 2.1. Ethical Statement

This study was conducted according to the guidelines of the Declaration of Helsinki and approved by the Ethical Committee and the Committee of Medicine (permit number: 2021-1-1213), and Animal Experimentation of the University of Tohoku Graduate School of Medicine (permit number: 2019MdLMO-134-03). Written informed consent was obtained from all participants.

### 2.2. Patients

Serum samples were obtained from 16 CTCL patients (MF patch, one; MF plaque, six; MF tumor, four; peripheral T-cell lymphoma-NOS, three; erythrodermic MF, one; folliculotropic MF, one; primary cutaneous anaplastic large cell lymphoma, one) who had not undergone systemic treatment and who visited Tohoku University Hospital between 2016 and 2020 and were diagnosed with CTCL. Serum samples were also obtained from 16 healthy control (HC) subjects who visited our hospital for the removal of benign tumors. Skin samples were obtained from 14 MF patients who had not undergone systemic treatment (patch stage, five; plaque stage, five; tumor stage, four) and from five healthy controls. Skin samples were obtained between 2007 and 2022 and stored as formalin-fixed, paraffin-embedded tissues until use.

### 2.3. Enzyme-Linked Immunosorbent Assay (ELISA)

ELISA kits for human HA, CXCL9, CXCL10, CXCL11, CCL17, and CCL22 (R&D Systems, Minneapolis, MN, USA) were used according to the manufacturer’s instructions. To measure serum levels of HA, serum samples from patients with cutaneous T-cell lymphoma and healthy control (HC) subjects were obtained and stored at −20 °C until use. Expression of CXCL9, CXCL10, CXCL11, CCL17, and CCL22 by human M2 macrophages with or without HA stimulation was measured using cell culture supernatant that was stored at −20 °C until use. Absorbance was measured at 450 nm. Concentrations were determined from standard curves.

Mouse tissue HA was measured using an ELISA (Purple-Jelley Hyaluronic Acid Assay Kit, Biocolor, Carrickfergus, UK) according to the manufacturer’s instructions. This kit allowed for both the extraction and measurement of HA from tissue samples. Absorbance was measured at 655 nm. Concentrations were determined from a standard curve.

### 2.4. Immunohistochemistry and Immunofluorescence Staining

Immunohistochemistry analysis was performed on formalin-fixed, paraffin-embedded skin sections using a Vectastain Elite ABC kit (Vector Laboratories, Burlingame, CA, USA) and antibodies against HAS1 (Merck, Darmstadt, Germany), HAS2 (Thermo Fisher Scientific, Waltham, MA, USA), HAS3 (Thermo Fisher Scientific), HYAL1 (Thermo Fisher Scientific), HYAL2 (Proteintech, Rosemont, IL, USA), and CEMIP2 (Thermo Fisher Scientific). Heat-mediated antigen retrieval was performed using VECTASTAIN Antigen Retrieval Solution pH 6 (Vector Laboratories). Samples were incubated with primary antibody overnight at 4 °C and then incubated with secondary antibody for 30 min at room temperature. Peroxidase activity was detected using 3,3′-diaminobenzidine (FUJIFILM Wako Pure Chemical Corporation, Osaka, Japan). Mayer’s hematoxylin was used for counterstaining. Skin samples were also subjected to hematoxylin-eosin staining.

To visualize HA, an immunofluorescence assay targeting HA-binding protein was performed using Hyaluronic Acid Binding Protein (HABP), Bovine Nasal Cartilage, Biotinylated (Merck), and Streptavidin, Alexa Fluor 488 conjugate (Thermo Fisher Scientific).

Two independent dermatologists performed evaluations for immunohistochemistry and immunofluorescence assays (T. Ikawa and T. Fujimura). The following grading system was used for staining intensity: 0, no staining; 1, slight staining; 2, moderate staining; 3, strong staining; 4, very strong staining. Exposure times are aligned for all specimens for observation.

### 2.5. RNA Isolation and Quantitative Reverse Transcription (qRT)–Polymerase Chain Reaction (PCR)

RNA was isolated from cells or tissues using the RNeasy mini kit (Qiagen, Hilden, Germany) and subjected to quantitative reverse transcription (qRT)-PCR using THUNDERBIRD SYBR qPCR Mix (TOYOBO, Osaka, Japan) on a Quantstudio 3 PCR System (Thermo Fisher Scientific) in duplicate. Dissociation analysis was performed for each primer pair to verify specific amplification. mRNA levels of target genes were examined and normalized to the mRNA level of *GAPDH*. The sequences of primers are summarized in Supplementary Tables S1 and S2.

### 2.6. Extraction of HA from Murine Skin Tissue and HA Electrophoresis

HA was extracted from mouse skin tissue using a Purple-Jelley Hyaluronic Acid Assay Kit (Biocolor) and then subjected to agarose gel electrophoresis on a 1% gel. HA at 5–15 μg was loaded into each well. Electrophoresis was performed at a constant voltage of 25 V for 0.5 h and then 50 V for 50 min. Select-HA HiLadder (Echelon Biosciences, Salt Lake City, UT, USA), select-HA LoLadder (Echelon Biosciences), and positive controls (sodium hyaluronate 2000 kDa, 250 kDa, and 50 kDa; all from Echelon Biosciences) were run alongside samples. Immediately after the run, the gel was placed in approximately 500 mL of 0.005% Stains-All (Santa Cruz Biotechnology, Dallas, TX, USA) in 50% ethanol and stained overnight with protection from light. For destaining, the gel was placed in 70% ethanol for approximately 6 h with several changes of the solution. Final destaining was performed by placing the gel under indoor lighting until the residual background became clear. The gel was scanned using ChemiDoc MP (Bio-Rad, Hercules, CA, USA) with a Cy5.5 filter. Bands were analyzed using Image Lab software (version 4.1, Bio-Rad).

### 2.7. Tumor Cell Inoculation and Bexarotene Treatment

EL4 T-cell lymphoma cells (American Type Culture Collection, Manassas, VA, USA; 100 μL of 2 × 10^6^ cells/mL) were subcutaneously injected into the skin on the back of each mouse on Day 0. For therapeutic experiments, 0.1 mg of bexarotene (Tokyo Chemical Industry, Tokyo, Japan) was intraperitoneally injected on Day 14, and the tumors were harvested on Day 16. Bexarotene was dissolved in dimethyl sulfoxide (Sigma-Aldrich, St. Louis, MO, USA) at a concentration of 10 mg/mL. A 10% solution of bexarotene was then mixed with 90% corn oil (Sigma-Aldrich) to a final concentration of 1 mg/mL. The vehicle was administered to the no-treatment group.

To evaluate the effect of LMWHA on tumor proliferation in vivo, the tumor-inoculated site was injected every day with 100 μL of 1 mg/mL LMWHA (250 kDa; Echelon Biosciences, Salt Lake City, UT, USA) dissolved in phosphate-buffered saline. The size of established tumors was measured using calipers, and tumor volume was estimated using the following formula: π/6 × length × width.

### 2.8. Measurement of Tumor Cell Proliferation

The proliferation of EL4 and HuT78 (American Type Culture Collection) cells was assessed by counting cells using a Countess Automated Cell Counter (Thermo Fisher Scientific) and the 3-(4,5-dimethylthiazol-2-yl)-2,5-diphenyltetrazolium bromide (MTT) method with CellTiter 96 Non-radioactive cell proliferation assay (Promega, Madison, WI, USA) according to the manufacturer’s instructions.

Lymphoma cells were serum-starved overnight in RPMI-1640 medium with 2% fetal bovine serum (FBS) and then seeded into a 24-well plate at 0.5 × 10^5^ cells/well on Day 0. The cells were cultured in 1 mL of RPMI-1640 with 2% FBS with or without HA. HA consisted of 1 mg/mL of LMWHA or HMWHA (250 kDa and 2000 kDa each; both from Echelon Biosciences). The number of total and live cells was determined from Days 1 to 5. Due to rapid cell proliferation, 1 mL of medium with the same concentration of HA was added on Day 3. Cells for MTT assay were likewise cultured in 96-well plates at 0.5 × 10^4^ cells/well at the start, and the assay was performed 5 days later.

### 2.9. Induction of M2 Macrophages from Human Peripheral Blood Mononuclear Cells (PBMCs)

To clarify the relationship between HA and tumor-associated macrophages (TAMs), human monocytes collected from PBMCs were differentiated into M2 macrophages. Human PBMCs were isolated from whole peripheral blood obtained from four HC subjects using SepMate-50 (STEMCELL Technologies, Vancouver, BC Canada) and Lymphoprep (PROGEN, Heidelberg, Germany). The collected PBMCs were treated with anti-CD14 microbeads (Miltenyi Biotech, Bergisch Gladbach, Germany) and positively selected using a MACS cell separation system (Miltenyi Biotech). Separated CD14-positive monocytes were cultured in a RPMI-1640 medium with 10% FBS and stimulated with 100 ng/mL of recombinant human macrophage colony-stimulating factor (Peprotech, Rockey Hill, NJ, USA) for 6 days. Cells that adhered to the bottom surface were regarded as macrophages. The cells were then incubated with recombinant human IL-4 (Peprotech) for another 2 days. Finally, 1 mg/mL of LMWHA or HMWHA (250 kDa and 2000 kDa each; both from Echelon Biosciences) was added to the induced-M2 macrophages, and the cells were incubated for another 24 h.

### 2.10. Tube Formation Assay

Human dermal microvascular endothelial cells (HDMECs) (Lonza, Zulich, Switzerland) were cultured to near confluence and serum-starved overnight. Then, μ-slides (ibidi, San Jose, CA, USA) were coated with 10 μL of growth factor-reduced Matrigel (Corning, Durham, NY, USA). After the gel was solidified, the cells were trypsinized and seeded into each well at 1 × 10^4^ cells per well and incubated in 50 μL of endothelial basal medium including 1% FBS for 6 h with or without HA (1 mg/mL of LMWHA 250 kDa or HMWHA 2000 kDa). Finally, the cells were treated with calcein AM (Dojindo, Kumamoto, Japan) before observation. Photographs were taken using a BZ-X800 (KEYENCE, Tokyo, Japan). The number of meshes and total tube length were calculated using ImageJ software version 1.54g (US National Institutes of Health, Bethesda, MD, USA).

### 2.11. Immunoblotting

To evaluate the effect of HA, semi-confluent normal human dermal fibroblasts (NHDFs) (American Type Culture Collection) were serum-starved overnight in a serum-free medium and stimulated with 1 mg/mL of LMWHA or HMWHA (250 kDa and 2000 kDa each; both from Echelon Biosciences) for 24 h. NHDFs and HuT78 cells were serum-starved overnight and then cultured with 100 ng/mL of bexarotene for the next 24 h to assess the effect of bexarotene stimulation. Whole-cell lysates were prepared using RIPA buffer (Thermo Fisher Scientific). Samples were subjected to sodium dodecyl sulfate-polyacrylamide gel electrophoresis (Thermo Fisher Scientific) and immunoblotting with anti-HAS1 antibody (Merck), anti-HAS2 antibody (Thermo Fisher Scientific), anti-HAS3 antibody (Thermo Fisher Scientific), anti-CTGF antibody (Abcam, Cambridge, UK), anti-CEMIP antibody (Thermo Fisher Scientific), or anti–β-actin antibody (Santa Cruz Biotechnology), followed by horseradish peroxidase-conjugated anti-mouse or anti-rabbit IgG secondary antibody (Cell Signaling Technology, Danvers, MA, USA). Protein bands were visualized by chemiluminescence using LumiGLO reagent (Cell Signaling Technology) and scanned using an ImageQuant LAS4000mini (Cytiva, Marlborough, MA, USA). The density of each band was quantified using ImageQuant TL software version 8.2 (Cytiva).

### 2.12. RNA Sequencing (RNA-Seq)

Sample preparation, sequencing, and alignment: Mouse tumor tissues treated with bexarotene or vehicle were compared. Tumor inoculation and bexarotene administration were performed as explained in ‘Tumor inoculation and bexarotene treatment’. RNA was extracted from tumor-inoculated mouse skin using an RNeasy mini kit (Qiagen) according to the manufacturer’s instructions. cDNA libraries for RNA-seq were prepared using a NEBNext Ultra II Directional RNA Library prep kit for Illumina (New England Biolabs, Ipswich, MA, USA). Briefly, sequencing was performed on the Illumina platform. Approximately 70 million reads per sample were uniquely aligned to the *Mus musculus* genome (GRCm39.104) using Hisat2 (version 2.1.0). HTSeq (version 0.6.1) was then used to estimate gene and isoform expression levels from the paired-end clean data.

Differential expression analysis and data visualization: Gene read count data were analyzed using the DESeq2 Bioconductor package (version 1.63). Estimates of dispersion and logarithmic fold-change incorporated data-driven prior distributions. The padj of genes was set to <0.05 to detect those that were differentially expressed. A heat map was generated using Heatmapper (http://www.heatmapper.ca/ (accessed on 2 September 2023)), and a volcano plot was generated using SRplot (http://www.bioinformatics.com.cn/srplot (accessed on 3 September 2023)). The raw data were deposited in the Gene Expression Omnibus database under accession number GSE248641.

### 2.13. Chromatin Immunoprecipitation (ChIP) Assay

ChIP assays were conducted on NHDFs using an EpiQuik ChIP kit (Epigentek, Farmingdale, NY, USA). Briefly, cells were treated with 1% formaldehyde for 10 min, and cross-linked chromatin was prepared and sonicated to an average size of 300–500 bp. DNA fragments were immunoprecipitated using an anti-RXRα antibody (Cell Signaling Technology) or normal rabbit IgG at 4 °C. After reversal of cross-linking, the immunoprecipitated chromatin was amplified by PCR of the specific region of the target genomic locus. Putative RXRα binding sites in the *HAS1*, *HAS2*, and *HAS3* promoters were predicted using JASPAR (https://jaspar.genereg.net/ (accessed on 26 June 2023)). Primers that amplify fragments of the *HAS1*, *HAS2*, and *HAS3* promoters are shown in Supplementary Table S1. The amplified DNA products were resolved by agarose gel electrophoresis to confirm RXRα binding. RXRα binding was quantified by calculating the enrichment score from the fold-increase in Ct value of the anti-RXRα antibody relative to that of rabbit IgG according to qRT-PCR.

### 2.14. Mice and Cells

Eight-week-old female wild-type C57BL/6JJmsSlc mice were used in the study. All mice were housed with three to six animals per cage under specific pathogen-free conditions and maintained on a 12-h light/dark cycle at a constant temperature (20–22 °C) and humidity (45–55%), with ad libitum access to food and water. All injections were conducted under inhalation isoflurane anesthesia. Upon completion of the experiment, the mice were euthanized by cervical dislocation. No inclusion or exclusion criteria were applied to the mice used in the study.

NHDFs, HDMECs, EL4 mouse lymphoma cells, and HuT78 human cutaneous lymphoma cells were used in this study. Cells were cultured at 37 °C with 5% CO_2_. NHDFs were cultured in Eagle’s Minimum Essential Medium with 10% fetal bovine serum (FBS) (Sigma-Aldrich, St. Louis, MO, USA), and HDMECs were cultured in endothelial Basal Medium-2 supplemented with the Endothelial Cell Growth Medium-2 Bullet Kit (Lonza). EL4 and HuT78 cells were cultured in RPMI-1640 medium (Thermo Fisher Scientific) containing 10% FBS. Cell culture inserts with 3-μm pore size (Corning) were used for coculture experiments.

### 2.15. Statistical Analysis

Statistical significance was evaluated using the Mann–Whitney *U*-test for two-group comparisons, and the Kruskal–Wallis test, followed by Dunn’s multiple comparison test, was used for multiple comparisons. Statistical significance was defined as *p* < 0.05. Statistical analyses were performed using GraphPad Prism 9 software (GraphPad Software, San Diego, CA, USA). Data are expressed as the mean ± SEM.

## 3. Results

### 3.1. HA Is Overexpressed in CTCL

**Figure 1:**
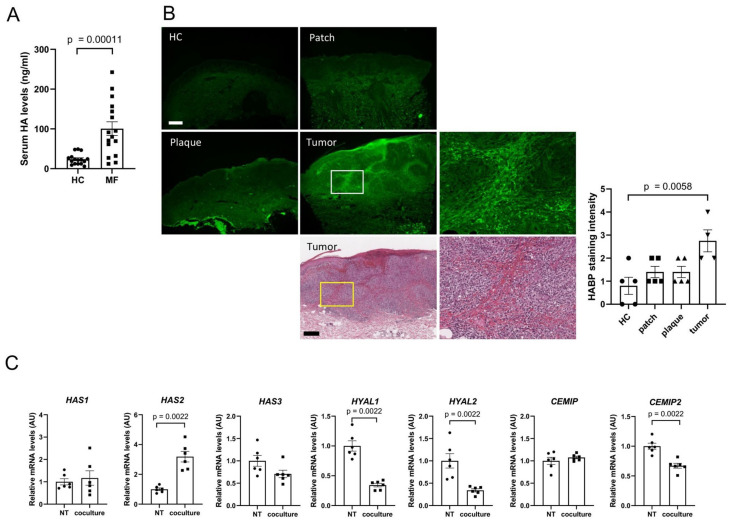

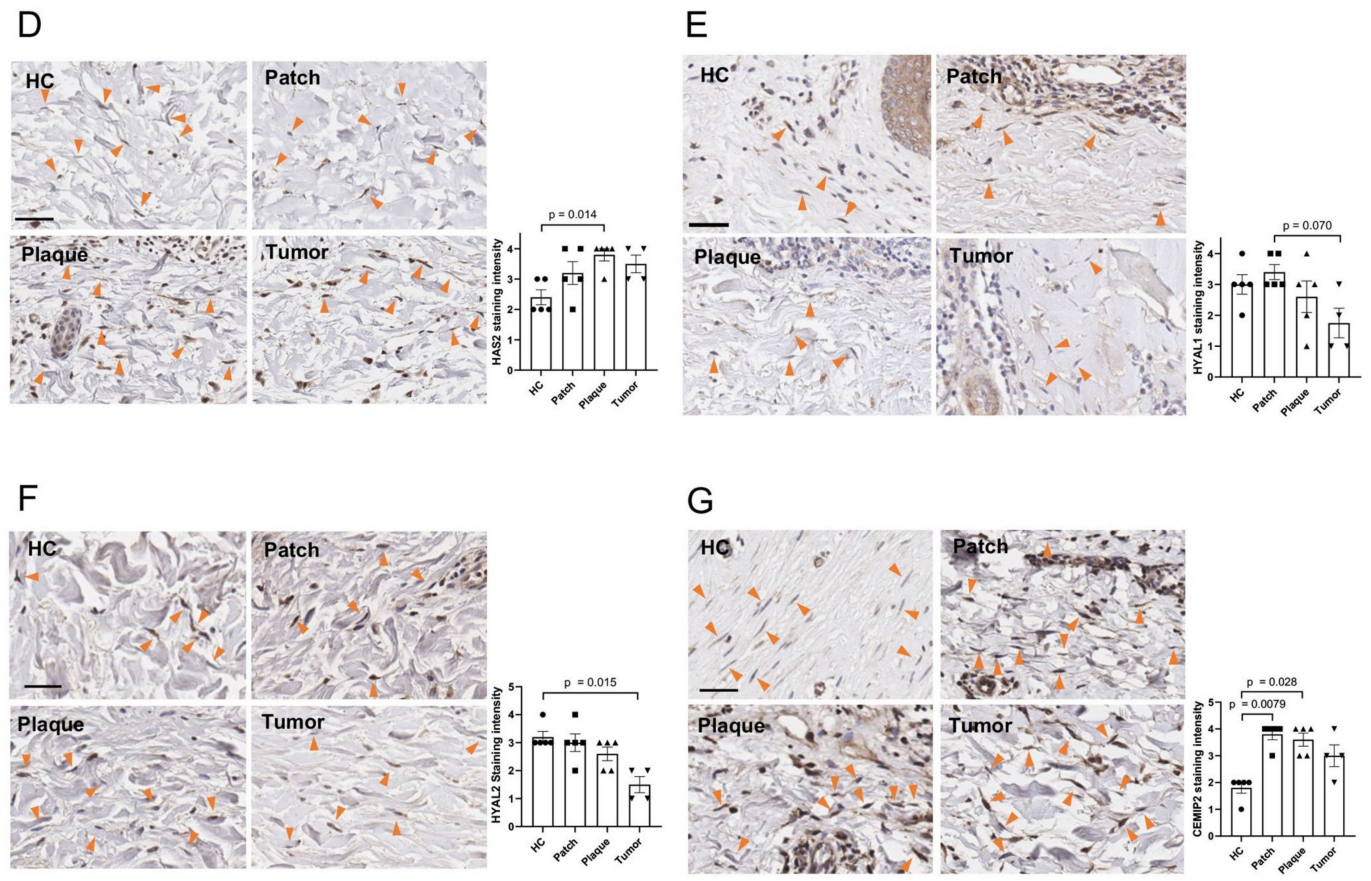
HA overexpression in CTCL. (**A**) Serum HA levels of CTCL patients were compared with those of HCs (n = 16 each). (**B**) Immunofluorescent staining of HABP on MF lesion and HC skin sections (n = 5 for HC, patch, and plaque; n = 4 for tumor). Magnified images indicated by squares are shown on the right. Bar = 300 µm. (**C**) mRNA expression of NHDFs cocultured with HuT78 cells (n = 6 each). (**D**–**G**) Immunohistochemistry analysis of HAS2, HYAL1, HYAL2, and CEMIP2 expression in MF lesion and HC skin sections (n = 5 for HC, patch, and plaque; n = 4 for tumor). Fibroblasts are indicated by arrowheads. Bar = 50 µm. Representative images are shown. HC, healthy control; CTCL, cutaneous T-cell lymphoma; HABP, hyaluronic acid binding protein; NT, no treatment; AU, arbitrary unit.

Serum HA levels in CTCL patients and HCs were measured using an enzyme-linked immunosorbent assay (ELISA) (Figure 1A). Serum HA levels were significantly higher in CTCL patients than in HCs. Immunofluorescence staining of HA-binding protein confirmed significantly higher HA expression in lesional skin sections of MF patients in the tumor stage (Figure 1B). HA accumulation was noted particularly around the tumor nests. Tumor cell staining intensity was positive but weak.

### 3.2. Lymphoma Cells Activate Fibroblasts, Resulting in Accumulation of HA

Fibroblasts are the primary source of HA in the skin. To simulate the skin invaded by lymphoma cells in vitro, NHDFs were cocultured for 24 h with HuT78 cells using cell culture inserts. Lymphoma cell lines were spread inside the insert, and fibroblasts were spread on a plate outside the insert. *HAS2* expression was significantly higher in NHDFs, whereas expression of *HYAL1*, *HYAL2*, and *CEMIP2* was significantly reduced (Figure 1C). Immunohistochemistry analysis was then carried out to examine the expression of HAS2, HYAL1, HYAL2, and CEMIP2 proteins (Figure 1D–G). Compared with HCs, HAS2 expression was significantly elevated in fibroblasts of plaque specimens from patient specimens. A tendency toward decreased HYAL1 expression was noted in the tumor stage compared with the patch stage. HYAL2 expression was significantly lower in tumor lesions compared with HC samples. CEMIP2 expression was significantly higher in MF skin than HC skin (Figure 1G), in contrast to the results of in vitro mRNA expression analysis (Figure 1C). The above data indicate that HA expression in MF lesional skin is increased due to increased HAS2 expression and decreased HYAL1 and HYAL2 expression. Enhanced CEMIP2 expression could result in a local increase in levels of LMWHA.

### 3.3. Lymphoma Cells Also Contribute to Local HA Accumulation

**Figure 2:**
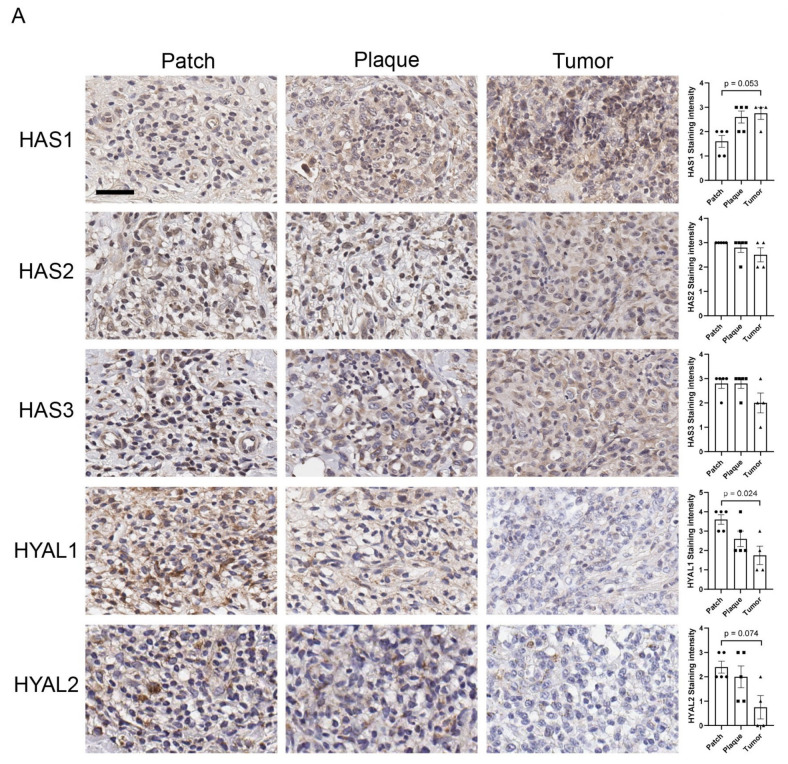

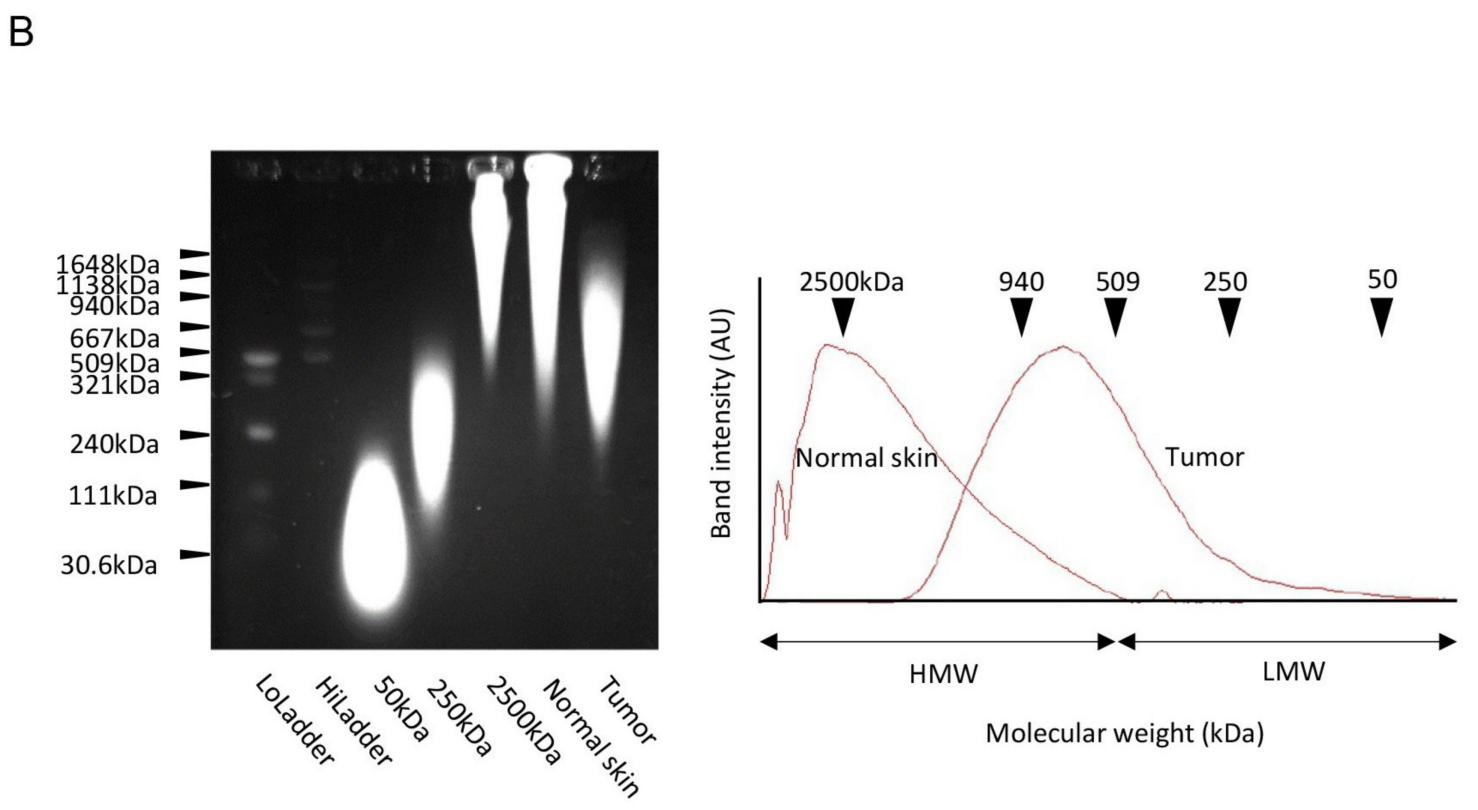
Contribution of tumor cells to HA accumulation and molecular weight distribution of HA from tumor-inoculated mouse skin. (**A**) Immunohistochemistry analysis of HAS1, HAS2, HAS3, HYAL1, and HYAL2 expression on tumor cells from MF patient skin sections (n = 5 for patch, and plaque; n = 4 for tumor). Bar = 50 µm. Representative images are shown. (**B**) Left panel: agarose gel electrophoresis of HA from normal murine skin and skin inoculated with EL4 cells. Right panel: graphical illustration of molecular weight distribution of HA in normal skin and tumor-inoculated skin. A representative image is shown. HMW; high-molecular-weight; LMW, low-molecular-weight.

HA production was also evaluated by immunohistochemistry analysis of HAS1, HAS2, HAS3, HYAL1, and HYAL2 expression on tumor cells (Figure 2A). HAS1 expression was significantly higher in the tumor stage compared to the patch stage, whereas expression of HAS2 and HAS3 was moderate to strong in all disease stages. HYAL1 and HYAL2 expression was significantly reduced in the tumor stage. High HAS1 expression in conjunction with low HYAL1 and HYAL2 expression was observed in the tumor stage, which resulted in the accumulation of HA around tumor cells. High HAS2 and HAS3 expression was maintained throughout the disease course. These data indicate that tumor cells are also a source of HA accumulation.

### 3.4. Analysis of the Molecular Weight Distribution of Tumor-Invaded Skin Indicates a Shift Toward LMWHA

HA was extracted from normal murine skin and EL4-inoculated skin with tumors. The result of agarose gel electrophoresis analysis of the specimens is shown in the left panel of Figure 2B. The band distributions of normal and tumor-affected skin are shown in the line graph of the right panel of Figure 2B. The molecular weight distribution of HA from normal skin was in the high range, whereas the molecular weight distribution of HA in tumor-affected murine skin tissue appeared to be in the low range, indicating that tumor-affected skin harbors more LMWHA than HMWHA.

### 3.5. HA Affects Tumor Cell Proliferation Both In Vivo and In Vitro

**Figure 3:**
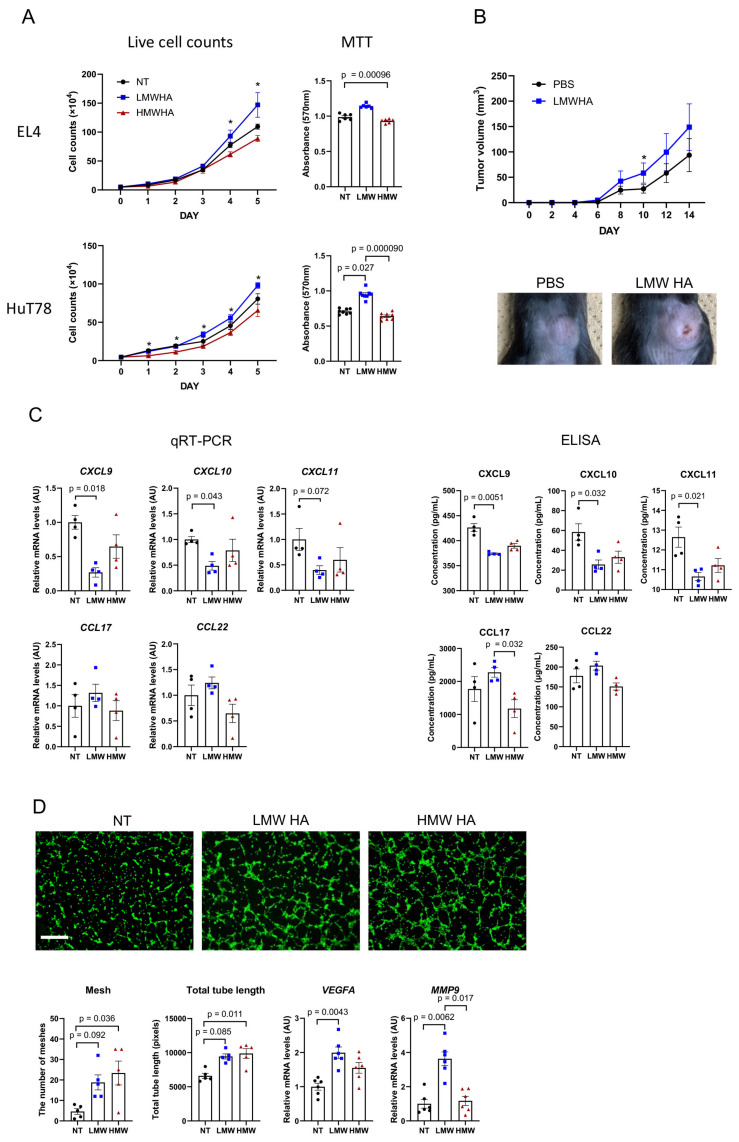
HA affects tumor cell proliferation and the CTCL microenvironment. (**A**) Proliferation of EL4 and HuT78 cells in vitro in the presence of LMWHA and HMWHA. The left graph shows the number of live EL4 and HuT78 cells (n = 4 each). The right panel shows the results of MTT proliferation assays (n = 6 each). The MTT results refer to cells measured five days after treatment. Asterisks indicate *p* < 0.05 in comparing LMWHA and HMWHA. (**B**) Change in tumor volume under LMWHA stimulation in vivo. The asterisk indicates *p* < 0.05. Representative images of tumor size at day 10 are shown. (**C**) Expression of Th1 and Th2 chemokines by M2 macrophages with HA stimulation (n = 4 each). (**D**) Tube formation assay performed with HDMECs treated with LMWHA or HMWHA. Representative images are shown (n = 5 each). Bar = 300 µm. AU, arbitrary unit; NT, no treatment; LMW, low-molecular-weight; HMW, high-molecular-weight.

The effect of HA on tumor cells was evaluated by adding 1 mg/mL of LMWHA or HMWHA to the cell culture medium and then counting the live cells for five consecutive days. The addition of LMWHA significantly increased the proliferation of EL4 and HuT78 cells, whereas the addition of HMWHA decreased the proliferation of these cells (Figure 3A). Similar results were obtained using the MTT cell proliferation assay (Figure 3A). To confirm that this result was reproducible in vivo, mice were injected with LMWHA every day in the same location where tumor cells were inoculated. A significant difference was noted on day 10 (Figure 3B). These results suggest that LMWHA stimulates tumor proliferation in vivo, especially in the initial stages of tumor formation.

### 3.6. HA May Enhance M2 Polarization of Tumor-Associated Macrophages (TAMs)

TAMs are immune cells that contribute to tumorigenesis by supporting the tumor microenvironment [10,11]. Because TAMs exhibit an M2-like phenotype [10,11,12], we developed M2 macrophages from peripheral blood mononuclear cells obtained from HCs. The M2 macrophages were stimulated with LMWHA and HMWHA to assess the effects on TAMs. qRT-PCR analysis indicated that LMWHA significantly reduced the expression of the T-helper 1 (Th1) chemokines *CXCL9*, *CXCL10*, and *CXCL11* compared with no treatment (Figure 3C). The results were validated at the protein level using an ELISA assay with cell culture supernatant. Compared with HMWHA stimulation, LMWHA stimulation also significantly increased the expression of the Th2 chemokine CCL17 at the protein level (Figure 3C). These results suggest that LMWHA enhances the M2 polarization of TAMs, thereby supporting an immunosuppressive microenvironment.

### 3.7. HDMECs Exhibit Increased Tube Formation When Exposed to LMWHA and HMWHA

LMWHA is known to stimulate angiogenesis, whereas HMWHA inhibits angiogenesis [13]. Skin-derived endothelial cells (i.e., HDMECs) were used in this study in order to simulate angiogenesis in the skin, in contrast to previous studies that used human umbilical vein endothelial cells. Both LMWHA and HMWHA induced tube formation (Figure 3D). In addition, compared with HMWHA, LMWHA increased the mRNA expression of *VEGFA* and *MMP9* in HDMECs, suggesting they are associated with different mechanisms of angiogenesis. These results also suggest that in the presence of excessive amounts of HA in the skin, skin-derived endothelial cells stimulate angiogenesis, regardless of HA molecular size.

### 3.8. LMWHA Induces the Expression of HAS3 and Extracellular Matrix Components in NHDFs

**Figure 4:**
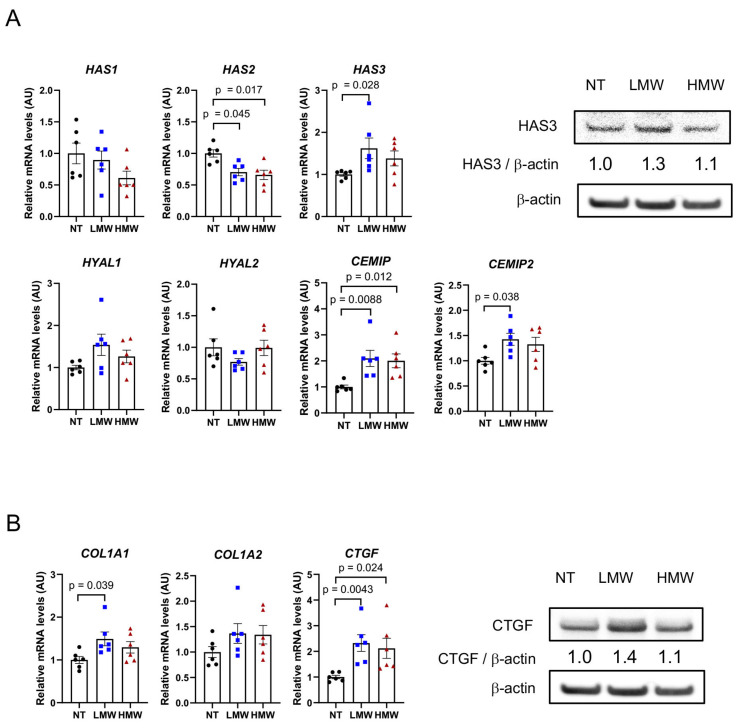
Response of NHDFs to LMWHA. (**A**) mRNA expression of HA-producing and depolymerizing molecules in NHDFs stimulated with LMWHA or HMWHA (n = 6 each). HAS3 expression is shown at the protein level (n = 4 each). (**B**) NHDFs were similarly treated with HA, and the mRNA expression of extracellular matrix-related molecules was measured (n = 6 each). The result of immunoblotting of CTGF is shown. AU, arbitrary unit; NT, no treatment; LMW, low-molecular-weight; HMW, high-molecular-weight. Original western blots are presented in Figure S1.

We also assessed the effect of HA on NHDFs (Figure 4). Serum-starved NHDFs were stimulated with 1 mg/mL of HA for 24 h. In NHDFs treated with LMWHA, the mRNA expression of *HAS3*, *CEMIP*, and *CEMIP2* was significantly higher than in NHDFs with no treatment (NT) (Figure 4A). Increased expression of HAS3 protein was demonstrated by immunoblotting (Figure 4A and Figure S1). Expression of the *HAS2* gene, which encodes HMWHA, was decreased instead. Increased levels of LMWHA may stimulate NHDFs to secrete more LMWHA into the extracellular space. The mRNA expression of *CEMIP* was also increased by HMWHA treatment, suggesting that excessive amounts of HA of any size around the cells enhance HA degradation by NHDFs.

LMWHA also promoted the expression of *collagen type 1 alpha 1* (*COL1A1*) and *connective tissue growth factor* (*CTGF*) (Figure 4B and Figure S1). Immunoblotting analysis indicated an increase in CTGF expression only with LMWHA treatment. In the presence of elevated levels of HA, LMWHA may stimulate NHDFs to produce more extracellular matrix components, leading to the formation of a rigid extracellular matrix around tumor cells.

### 3.9. Bexarotene Decreases HA Production In Vivo in CTCL Model Mice

**Figure 5:**
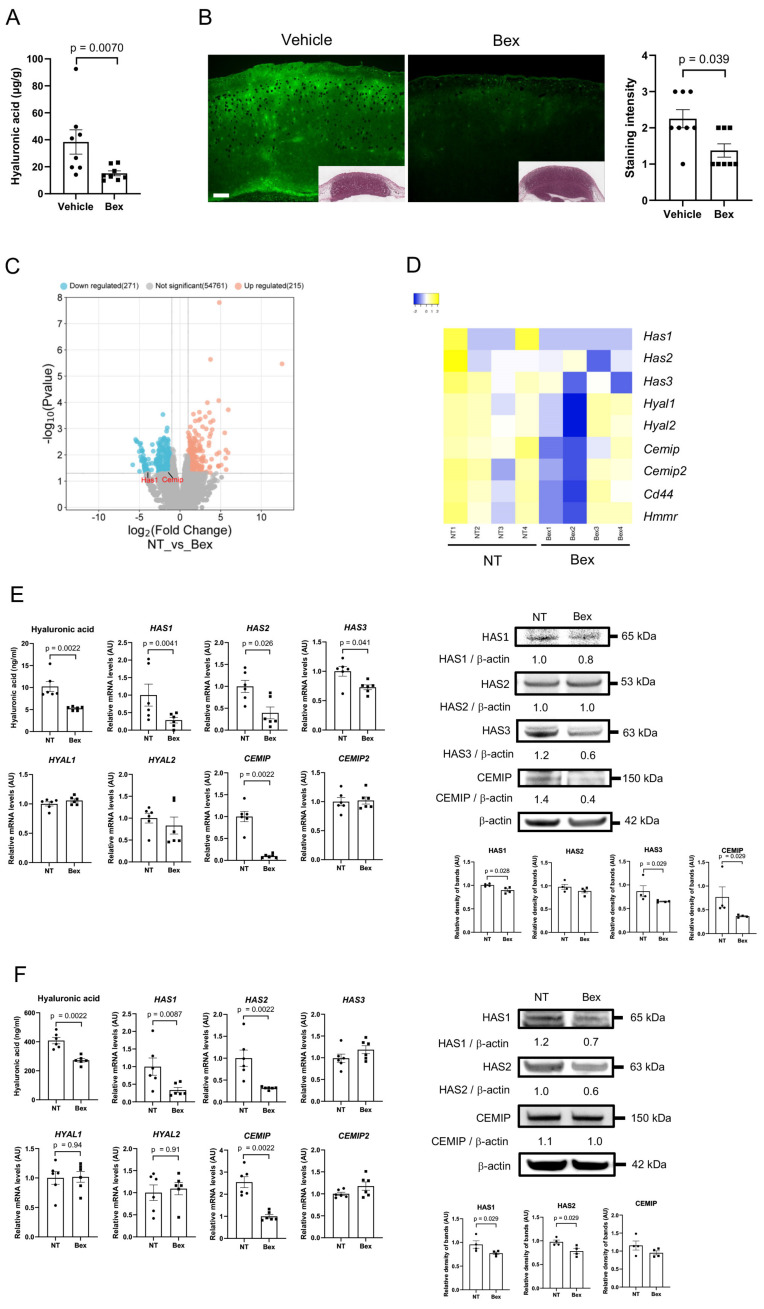
Bexarotene inhibits HA expression in vivo and in vitro. (**A**) Amount of HA extracted from mouse tumor tissue treated with or without bexarotene (n = 8 each). (**B**) Immunohistochemistry analysis of HABP expression in murine tumor skin sections treated with bexarotene (n = 8 each). Bar = 300 μm. (**C**) Volcano plot of RNA sequencing. Gene expression in bexarotene-treated tumor tissue specimens was compared with that of non-treated specimens (n = 4 each). Up-regulated genes are shown in blue, and down-regulated genes are shown in red. Above the horizontal solid line indicates *p* < 0.05. The fold-change cut-off was set at 2.0. (**D**) Heatmap of related gene expression based on log_2_ TPM. (**E**) HA concentration in cell culture supernatant and qRT-PCR and immunoblotting results in HuT78 cells treated with bexarotene or vehicle (n = 6 each). (**F**) HA concentration in cell culture supernatant and qRT-PCR and immunoblotting results of NHDFs treated with bexarotene or no treatment (n = 6 each). NT, no treatment; Bex, bexarotene; TPM, transcripts per kilobase million; AU, arbitrary unit. Original western blots are presented in Figures S2 and S3.

Intraperitoneal bexarotene administration significantly decreased the level of HA in tumor-inoculated mouse skin (Figure 5A). This result was confirmed by fluorescent immunohistochemistry analysis (Figure 5B). Tumor-inoculated mouse skin treated with bexarotene was compared with vehicle-treated skin using bulk RNA sequencing (Figure 5C,D). No statistically significant changes in mRNA expression of HA-related molecules were observed with bexarotene treatment, but a trend toward decreased expression was observed for *Has1* (*p* = 0.096) and *Cemip* (*p* = 0.092).

### 3.10. Bexarotene Suppresses HA Production by HuT78 Cells and NHDFs In Vitro

In vitro experiments were conducted to elucidate the mechanism of action of bexarotene. First, HuT78 cells were treated with 100 ng/mL of bexarotene (Figure 5E and Figure S2). The level of HA in the culture supernatant was reduced by 50% in cells treated with bexarotene. Bexarotene treatment also reduced mRNA expression of the *HAS1*, *HAS2*, *HAS3*, and *CEMIP* genes. Treated cells showed a decrease in HAS1 protein level relative to control cells, indicating a decrease in HMWHA content. Decreases in HAS3 and CEMIP expression were also confirmed, suggesting a reduction in LMWHA production by tumor cells.

NHDFs were then subjected to the same treatment (Figure 5F and Figure S3). The level of HA in the culture supernatant was significantly reduced by almost 50% in cells treated with bexarotene. As NHDFs produced a much higher amount of HA than HuT78 cells (Figure 5E,F), this reduction may have a significant impact on the skin. Bexarotene treatment also reduced mRNA expression of the *HAS1*, *HAS2*, and *CEMIP* genes. At the protein level, the expression of both HAS1 and HAS2 was decreased by bexarotene treatment. These results suggest that bexarotene can decrease excessive HMWHA production in NHDFs.

### 3.11. Bexarotene Decreases the Capacity of Retinoid X Receptor α (RXRα) to Bind the HAS1 and HAS2 Promoters

**Figure 6:**
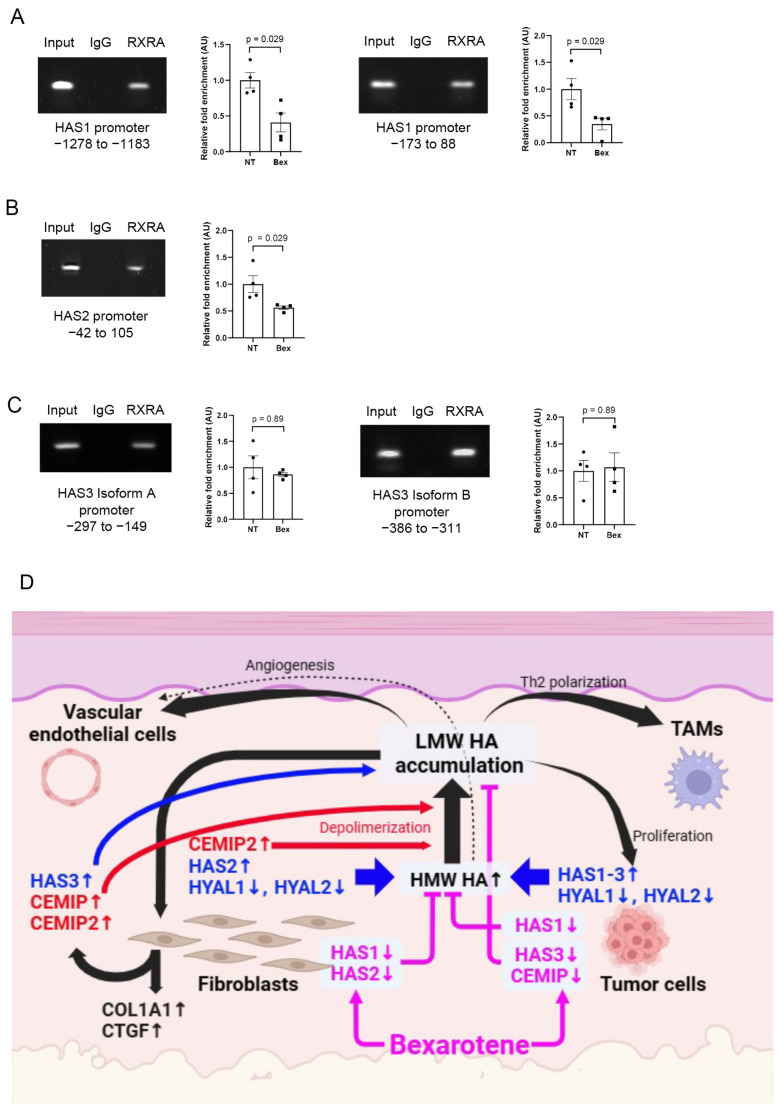
Bexarotene affects the DNA-binding capacity of retinoid X receptor α (RXRα). (**A**–**C**) RXRα bind to *HAS1*, *HAS2*, and *HAS3* promoter regions as demonstrated by chromatin immunoprecipitation. Enrichment analysis was performed to compare RXRα binding capacity under stimulation with bexarotene and with no treatment (n = 4 each). (**D**) Summary illustrating the relationship between CTCL, HA, and bexarotene. NT, no treatment; Bex, bexarotene.

We found that bexarotene reduced the expression of HAS1 and HAS2 in fibroblasts. To elucidate the mechanism by which bexarotene decreases HA production, we performed a chromatin immunoprecipitation assay on NHDFs (Figure 6A–C). Bexarotene is a retinoid-derived compound that binds exclusively to retinoid X receptors, which are intranuclear receptors with three subtypes: α, β, and γ [14,15]. Bexarotene binding induces a change in the conformation of RXRs and promotes binding to DNA as a dimer with another receptor partner, in turn activating the transcription of target genes. Because RXRα is a major receptor expressed in the skin [14,16], we examined the binding of RXRα to the *HAS1-3* promoters. The promoter region of *HAS1* has two possible binding sites for RXRα. The binding of RXRα to these sites was demonstrated by agarose gel electrophoresis (Figure 6A). Fold-enrichment analysis based on Ct score showed lower RXRα binding with bexarotene than NT.

The promoter of *HAS2* has only one possible binding site for RXRα, which exhibited decreased RXRα binding following bexarotene stimulation (Figure 6B). The promoters for *HAS3* isoforms A and B each have one possible binding site for RXRα, and electrophoresis demonstrated actual binding. However, the binding of RXRα to these promoters was not affected by bexarotene (Figure 6C). Based on the above data, bexarotene appears to reduce the capacity of RXRα to bind the promoters of *HAS1* and *HAS2*, thereby suppressing the expression of these genes.

## 4. Discussion

The skin as an organ contains over one-third of the HA in the body [17]. HA molecules turn over rapidly, with a half-life of 12–24 h in the skin and minutes in the bloodstream [18]. This extremely rapid turnover is supported by the delicate balance between HA production and degradation. It is particularly important to understand the two pathways of HA degradation in malignant tumors, one involving HAYL1/HYAL2 and the other CEMIP/CEMIP2. The former pathway digests HA within cells, leading to a decrease of HA in the extracellular matrix. Conversely, the latter pathway increases the amount of LMWHA in the extracellular matrix by depolymerizing extracellular HMWHA or expelling LMWHA to the extracellular space [3]. LMWHA is oncogenic because it inhibits Hippo signal activation and thereby activates yes-associated protein, which drives neoplastic transformation of cells [19]. Increased levels of LMWHA in malignant tumors [20] suggest that HAS3, CEMIP, and CEMIP2 function as key enzymes. It was recently reported that HMWHA also induces angiogenesis [21,22]. Huang et al. reported that HMWHA stimulates angiogenesis by upregulating the phosphorylation of Src, ERK, and AKT in endothelial cells [21]. Therefore, HA size-dependent angiogenesis may be tissue or context-dependent. However, the possibility that LMWHA produced by HA-degrading enzymes expressed by HDMECs may have promoted angiogenesis in this study cannot be ruled out.

Figure 6D provides a summary of the present study. Tumor cells express HAS1–3 throughout the disease course, whereas expression of HYAL1 and 2 decrease in the tumor stage, leading to HA accumulation. However, tumor cells produce much less HA than fibroblasts, as shown in Figure 1B and Figure 5E. Stimulation of fibroblasts by tumor cells results in increased expression of HAS2, decreased expression of HYAL1 and 2, and subsequently increase levels of HMWHA in the extracellular matrix. The simultaneous increase in CEMIP expression facilitates the production of LMWHA, and increased LMWHA levels promote tumor proliferation. LMWHA, in turn, promotes the production of more HAS3, CEMIP, and CEMP2 by fibroblasts. Fibroblasts around tumor nests may form rigid stroma with HA by promoting the expression of COL1A1 and CTGF, which can form a barrier that surrounds tumor cells and limits access to the tumor by immune cells [23]. This mechanism could also affect the efficacy of pharmacological agents, such as monoclonal antibody therapy [24]. LMWHA intensifies Th2 polarization of TAMs, further enabling tumor cells to evade the immune response and sustain the immunosuppressive function of TAMs [25]. Importantly, our data indicate that bexarotene reduces LMWHA and HMWHA accumulation by suppressing the expression of HAS1, HAS3, and CEMIP in tumor cells and HAS1 and HAS2 in fibroblasts.

HA-targeted therapies have been investigated at various levels due to the critical role of HA in cancer development and progression. Blocking the interaction with CD44 receptors has been extensively tested in mice, with promising results [26], but the efficacy seems modest in humans [27]. In contrast, there has been little research into therapies aimed at reducing HA production or targeting HA synthases. Only one agent that inhibits HA synthesis, 4-methylumbelliferone (4-MU), has been reported to date [28]. 4-MU exhibited promising effects in vitro against cells of various cancers, such as hepatocellular carcinoma, colorectal carcinoma, and breast carcinoma [29]. Nevertheless, 4-MU should be used carefully, as it can cause severe damage to the endothelium, accelerating atherosclerosis [26]. Thus, there is an urgent need to discover new substances that can suppress HA biosynthesis in tumor cells.

Bexarotene is an orally administered retinoid-derived synthetic compound recommended for patients with early-stage MF in combination with skin-directed therapy and for patients with advanced-stage disease with inadequate response to single-agent therapy [30]. Bexarotene exerts anti-cancer effects by inducing apoptosis and differentiation, preventing multidrug resistance, and inhibiting angiogenesis and metastasis [15]. Furthermore, bexarotene exhibits a diverse range of effects, modulating not only tumor cells but also the tumor microenvironment, including T cells [31], macrophages [32], and angiogenesis [33]. Therefore, we hypothesized that bexarotene may also affect HA metabolism. Indeed, bexarotene decreased the binding of the transcription factor RXRα to the *HAS1* and *HAS2* promoters, resulting in lower HA expression.

Bexarotene is a nuclear receptor that binds exclusively to RXRs, serving as a ligand-activated transcription factor [31]. Bexarotene is known to cause hypothyroidism as a side effect of lowered gene expression. Bexarotene also suppresses the activity of the thyroid-stimulating hormone beta-subunit gene promoter, causing central hypothyroidism [34]. In this study, the expression of HAS genes was decreased via a similar mechanism. Inhibition of the binding of RXRα to the *HAS1* and *HAS2* promoter regions led to a decrease in the expression of HAS1 and HAS2, resulting in lowered synthesis of HA by dermal fibroblasts, which are the main source of HA in the skin. A reduction in the supply of HMWHA eventually decreases the level of LMWHA. Reducing HA levels thus represents a newly described anti-tumor mechanism of bexarotene in the treatment of CTCL.

Our study revealed novel findings that shed light on possible applications of bexarotene in stroma-targeted cancer therapy. Solid malignant tumors usually develop HA-rich rigid stroma that block therapeutic medications from reaching tumor cells [35]. Bexarotene may thus have a positive effect on susceptible cancers when used in combination with other tumoricidal agents. Indeed, bexarotene has been tested beyond CTCL for the treatment of lung [36] and breast cancer [37], with ongoing clinical trials [38]. Further investigations to verify bexarotene’s efficacy will be our objective.

## 5. Conclusions

Collectively, the results of our study demonstrate that HA plays a role in the development of CTCL by affecting the tumor and its microenvironment (Figure 6D). Moreover, bexarotene exerts potential anti-tumor effects by reducing HA levels, thereby providing an additional therapeutic mechanism in cancer treatment. These findings suggest a novel therapeutic approach for cancer treatment that focuses on targeting HA. Moreover, our present study suggests that MF may be more indolent in older patients due to less HA and fewer active fibroblasts. To prove this hypothesis, further national wide cohort studies will be needed in the future.

## Data Availability

The data that support the findings of this study are available on request from the corresponding author, T.F. The data are not publicly available due to containing information that could compromise the privacy of research participants.

## Supplementary Materials

The following supporting information can be downloaded at: https://www.mdpi.com/article/10.3390/cancers17020324/s1, Table S1. Primer sequences used for human target genes and chromatin immunoprecipitation assay; Table S2. Primer sequences used for murine target genes; Figure S1. Raw data for Figure 4A,B; Figure S2. Raw data for Figure 5E; Figure S3. Raw data for Figure 5F.
