# Supplementary material for: Impact of Hyaluronic Acid on the Cutaneous T-Cell Lymphoma Microenvironment: A Novel Anti-Tumor Mechanism of Bexarotene
Source: Cancers (Basel). 2025 Jan 20;17(2):324. doi: 10.3390/cancers17020324 (PMC11764198; doi:10.3390/cancers17020324)
Supplement: Supplementary file 1 [file cancers-17-00324-s001.zip › cancers-3397722-supplementary.pdf]

Supplementary Materials: Impact of Hyaluronic Acid on the Cutaneous T-Cell Lymphoma Microenvironment: A Novel Anti-Tumor Mechanism of Bexarotene

Tetsuya Ikawa, Emi Yamazaki, Ryo Amagai, Yumi Kambayashi, Mana Sekine, Takuya Takahashi, Yoshihide Asano and Taku Fujimura *

Table S1. Primer sequences used for human target genes and chromatin immunoprecipitation assay.

HAS1 forward	5′- TCCACTGTGTATCCTGCATCAG -3′
HAS1 reverse	5′- TGTACCAGGCCTCAAGAAACTG -3′
HAS2 forward	5′- AAAGCTCGCAACACGTAACG -3′
HAS2 reverse	5′- TAATCCACACTTCGTCCCAGTG -3′
HAS3 forward	5′- TCACTGCATTTGCCTGCTTC -3′
HAS3 reverse	5′- ACAGCCAAAAACAGCCTCAG -3′
HYAL1 forward	5′- ACTGCAGCAATCACAAAGGC -3′
HYAL1 reverse	5′- GGCAACGTGCTTACTCTCAATG -3′
HYAL2 forward	5′- AGATGCTGCAGAAACGTGTG -3′
HYAL2 reverse	5′- ATCTTTGTCCTGCCAGTTGC -3′
CEMIP forward	5′- AGCCGATTGTTTTGCGAACC -3′
CEMIP reverse	5′- TCCGGCTGAATACCTTCATCAG -3′
CEMIP2 forward	5′- TCAAAGCCAAAAGCCACAGG -3′
CEMIP2 reverse	5′- TGCTTTGGCCATGCAGTTAC -3′
CXCL9 forward	5′- ATGTTCACCCAACCACATCC -3′
CXCL9 reverse	5′- TTCAAACTGCTTGGCTCACC -3′
CXCL10 forward	5′- GAAAGCAGTTAGCAAGGAAAGGT -3′
CXCL10 reverse	5′- GACATATACTCCATGTAGGGAAGTGA -3′
CXCL11 forward	5′- TACAGTTGTTCAAGGCTTCCCC -3′
CXCL11 reverse	5′- GCCACTTTCACTGCTTTTACCC -3′
CCL17 forward	5′- ACGAAGAAGAGCCACAGTGAG -3′
CCL17 reverse	5′- TTAATCTGGGCCCTTTGTGC -3′
CCL22 forward	5′- ATGGATCGCCTACAGACTGC -3′
CCL22 reverse	5′- CCTGAAGGTTAGCAACACCAC -3′
VEGFA forward	5′- TTTGGGAACACCGACAAACC -3′
VEGFA reverse	5′- ATCCCCAAAGCACAGCAATG -3′
MMP9 forward	5′- ATGCCTGCAACGTGAACATC -3′
MMP9 reverse	5′- AGAATCGCCAGTACTTCCCATC -3′
COL1A1 forward	5′- TTGACCAACCGAACATGACC -3′
COL1A1 reverse	5′- TTCAAGCAAGTGGACCAAGC -3′
COL1A2 forward	5′- TGCTGGCAAACATGGAAACC -3′
COL1A2 reverse	5′- TTATCGCCACGAATGCCTTG -3′
CTGF forward	5′- AGGCAGTTGGCTCTAATCATAGTTG -3′
CTGF reverse	5′- GAGGAGTGGGTGTGTGACGAG -3′
GAPDH forward	5′- ACCCACTCCTCCACCTTTGA -3′
GAPDH reverse	5′- CATACCAGGAAATGAGCTTGACAA -3′
HAS1 promoter-1 forward	5′- TGCTGCTCCATGTCTTTCAG -3′
HAS1 promoter-1 reverse	5′- AGCTGCCTCGTTCTTTTCTC -3′
HAS1 promoter-2 forward	5′- TGCTGCTCCATGTCTTTCAG -3′
HAS1 promoter-2 reverse	5′- AGCTGCCTCGTTCTTTTCTC -3′

HAS2 promoter forward	5′- TTAAGTTGGAGGAGGCAGAAGG -3′
HAS2 promoter reverse	5′- GCGCAGAATTGGGAGAAAAGTC -3′
HAS3 isoform A promoter forward	5′- CATTTTGGGGGCCTCTATTTGG -3′
HAS3 isoform A promoter reverse	5′- CAGCTACCTTGGCAAAGTGTG -3′
HAS3 isoform B promoter forward	5′- GGAAAGTTAACGACGCTTCAGG -3′
HAS3 isoform B promoter reverse	5′- CAATCGCCCTGGGACCAG -3′

Table S2. Primer sequences used for murine target genes.

Has1 forward	5′- ACACAGCTTTCAAGGCACTG -3′
Has1 reverse	5′- TCATCCAACACTCGCACAAG -3′
Has2 forward	5′- TATCTTGGCTGGTGCTGTGTAG -3′
Has2 reverse	5′- TGGTGCTCTTTTTGCTTCGC -3′
Has3 forward	5′- TGCATGGACGTAACAACAGC -3′
Has3 reverse	5′- TTTGGGCAACTCCAGAAAGC -3′
Hyal1 forward	5′- TCATGCCAGGCCATTAAAGC -3′
Hyal1 reverse	5′- TTCGCTGCACAAAAGAGCTG -3′
Hyal2 forward	5′- TGAAGGAATCTGTGGAACGC -3′
Hyal2 reverse	5′- TGCCAGTTTCGAACCCATAC -3′
Cemip forward	5′- TTTGAATGCAGTGCCCGATG -3′
Cemip reverse	5′- AGTTTGGTCATTGCCTTCCG -3′
Cemip2 forward	5′- TTGAGCAGTTTCTCCCGTTG -3′
Cemip2 reverse	5′- AGCACTTTCAAAGCCTGCTG -3′
Gapdh forward	5′- CGTGTTCCTACCCCCAATGT -3′
Gapdh reverse	5′- TGTCATCATACTTGGCAGGTTTCT -3′

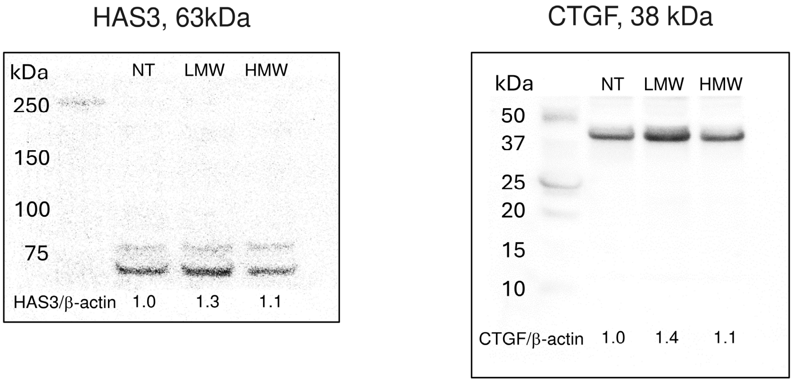

Figure S1. Raw data for Figure 4A and 4B.

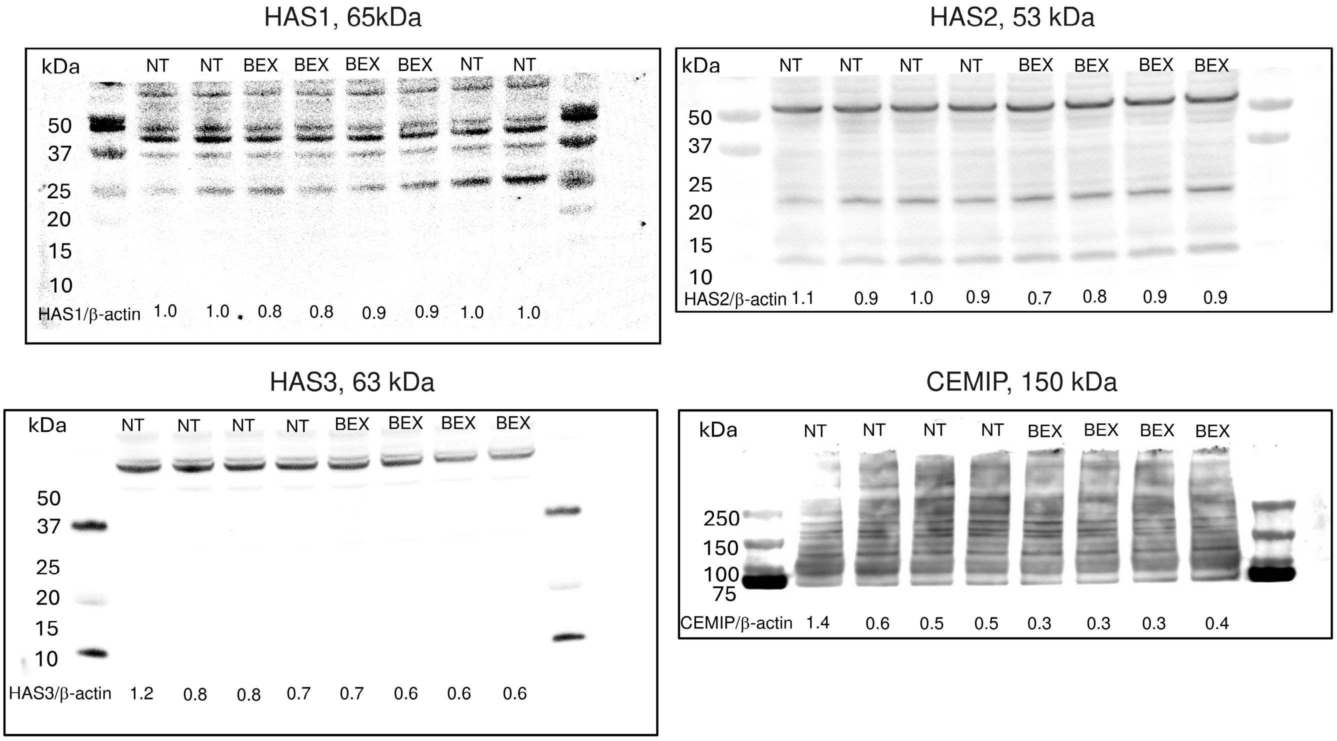

Figure S2. Raw data for Figure 5E.

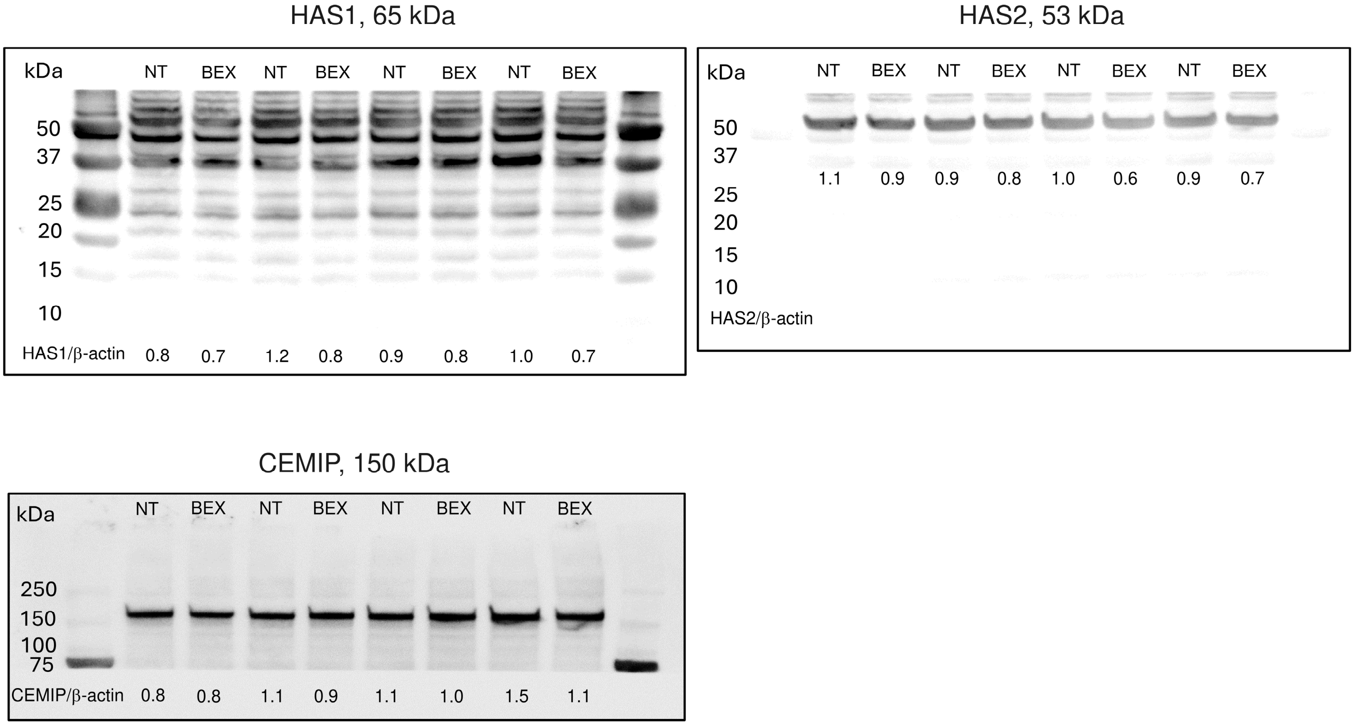

Figure S3. Raw data for Figure 5F.
